# Chemical and Mechanical Properties of Experimental Dental Composites as a Function of Formulation and Postcuring Thermal Treatment

**DOI:** 10.1155/2018/9845427

**Published:** 2018-03-15

**Authors:** Renata A. Esteves, Letícia C. C. Boaro, Flávia Gonçalves, Luiza M. P. Campos, Cecy M. Silva, Leonardo Eloy Rodrigues-Filho

**Affiliations:** ^1^School of Dentistry, University Federal of Pará, Rua Augusto Corrêa 01, 66075-110 Belém, PA, Brazil; ^2^School of Dentistry, University of Santo Amaro, Rua Professor Enéas de Siqueira Neto 340, 04829-300 São Paulo, SP, Brazil; ^3^University Ibirapuera, Av. Interlagos 1329, 04661-100 São Paulo, SP, Brazil; ^4^Nuclear and Energy Research Institute, University of São Paulo, Av. Lineu Prestes 2242, 05508-000 São Paulo, SP, Brazil; ^5^Department of Biomaterials and Oral Biology, School of Dentistry, University of São Paulo, Av. Lineu Prestes 2227, 05508-900 São Paulo, SP, Brazil

## Abstract

This study evaluated the influence of formulation and thermal treatment on the degree of conversion, fracture toughness, flexural strength, and elastic modulus of experimental composites. Six composites were analyzed at BisGMA : TEGDMA molar ratios of 1 : 1 and 7 : 3 with filler at 30, 50, and 70 wt%. The degree of conversion was analyzed by Fourier transform infrared spectroscopy, fracture toughness was measured using the single-edge notched beam, and flexural strength and elastic modulus were measured with the 3-point bend test. For all tests, one-half of the specimens received thermal treatment at 170°C for 10 min. Data were analyzed by the Kruskal-Wallis or ANOVA/Tukey's test (*α* = 5%). The 1 : 1 BisGMA : TEGDMA ratio showed higher properties than the 7 : 3 ratio. Although the material with 70% filler had a conversion lower than the one with 50%, it showed higher mechanical properties. The thermal treatment improved all properties in all materials. Therefore, the use of an equimolar ratio of BisGMA : TEGDMA can be paired with 70 wt% filler to design dental composites that possess increased advantageous physical and chemical properties. Furthermore, the simple and low-cost method of thermal treatment proposed for use in clinical dentistry has been shown to effectively improve the properties of all evaluated materials.

## 1. Introduction

Reconstructing dental elements with a significant loss of dental structure continue to be a challenge in oral rehabilitation because the absence of much of the crown structure may contraindicate performing direct procedures. Therefore, there is a need to use restorative materials that can reconstruct such structures, are biocompatible, and have satisfactory biomechanical and aesthetic properties, especially for use in areas under large masticatory forces [1].

Although the composites for both direct and indirect use generally have similar properties, the effects of variation in composition, especially in experimental composites, are being thoroughly studied [2–9]. Thus, it is known that the size, shape, distribution, composition, and concentration of the filler content, as well as the ratio between the main organic compounds that constitute the matrix, can affect the values of important properties, such as hardness, fracture toughness, diametral tensile strength, flexural strength, and elastic modulus [5, 7, 10–15]. For durability in treatment, it is of the utmost importance that resins demonstrate appropriate mechanical behavior, which is directly linked to the degree of conversion of the polymer matrix, the monomer composition, the fraction of inorganic filler, and the size and type of filler particles [5, 16, 17].

Given the need for these resins to exhibit excellent clinical performance, some studies have shown that it is possible to perform additional postcuring thermal treatment using equipment such as ovens, autoclaves, and special furnaces to improve their mechanical, physical, and chemical properties [18–21]. Published investigations have shown that the postcuring process of dental composites may improve properties such as flexural strength, wear resistance, fracture strength, and micro hardness [22–24]. However, a protocol for obtaining additional polymerization should be used without causing undesirable changes in the restorative material [19, 25].

Thus, a postcuring thermal treatment protocol was established for commercial composites, and it was found that the maximum gain of mechanical properties was achieved in temperature not coincident with the temperature at which the maximum degree of conversion was achieved; actually the maximum gain of mechanical properties was achieved at a lower temperature than that of the maximum degree of conversion. This result led to the assumption that, beyond the degree of conversion, it may be possible to achieve some gain in strength by stress relief [20]. It was also shown that both the monomer and inorganic fractions significantly affected the stresses generated by polymerization beyond the conversion [26].

Therefore, the aim of this study was to assess the influence of formulation and postcuring thermal treatment on chemical and mechanical properties of experimental composites, such as degree of conversion, fracture toughness, flexural strength, and elastic modulus. The null hypotheses were that formulation and postcuring thermal treatment would not affect the properties of experimental composites.

## 2. Materials and Methods

### 2.1. Preparation of Composites

In this experiment, 6 experimental composites with 2 resin matrices based on BisGMA (2,2-bis[p-(2′-hydroxy-3′-methacryloxypropoxy)phenylene]propane, ESSTECH, Essington, PA, USA) and TEGDMA (2-methyl-2-propenoic acid, ESSTECH) were used at ratios of 5 : 5 and 7 : 3 (in mol), respectively. Barium glass (average size 0.8 *μ*m) was used as a filler at concentrations of 30%, 50%, and 70% by weight. The composites were handled according to Table 1.

### 2.2. Thermal Treatment

Postcuring thermal treatment consisted of maintaining the specimens at 170°C for 10 minutes in an oven with a precisely controlled temperature (Orion 520, Fanem, São Paulo, Brazil) immediately after photoactivation. After postcuring thermal treatment, the samples were assigned according to the tests performed. For all factors studied, one-half of the specimens received thermal treatment, and the other half did not.

### 2.3. Degree of Conversion

The degree of conversion of the experimental composites (*n* = 5) was measured by Fourier transform infrared spectroscopy (FTIR, Vertex 70, Bruker Optik GmbH, Ettlingen, Germany). A spectrometer with the following specifications was used: an extended range KBr beam splitter, an InGaAs detector, 1 mm aperture, wavelength range from 4,000 cm^−1^ to 9,840 cm^−1^, and 6 cm^−1^ resolution. The specimens were fabricated using silicone molds with an 8 mm hole in the center, where the composite was placed and pressed between two glass slides. The mold-composite-glass set was positioned in the FTIR such that the reference laser beam and the IR source beam passed through the center of the specimen. Spectra were obtained for the unpolymerized, polymerized, and thermally treated materials. Each spectrum consisted of 2 scans. Photoactivation was performed for 20 seconds with a power density of 16 J/cm^2^ using an LED curing device (Elipar FreeLight 2, 3M ESPE, St. Paul, MN, USA). The curing power was measured using a radiometer before manufacturing all specimens (SDI LED Radiometer, Bayswater, Victoria, Australia). The area under the absorption peak for the vinyl bond located at 6,165 cm^−1^ was calculated using the software Opus v.6 (Bruker Optics, GmbH) for the unpolymerized and polymerized material and used to calculate the degree of conversion according to the following equation:

(1) $$\begin{array}{c} DC=100\times \left(\;1-\frac{\text{polymerized}}{\text{unpolymerized}}\;\right). \end{array}$$

### 2.4. Fracture Toughness (*K*_IC_)

The fracture toughness of the experimental composites was analyzed using the single-edge notched beam (SENB) method, which consists of assessing a specimen based on a defect (notch) present in the sample. A small stainless steel split mold was used to prepare specimens measuring 10 × 2 × 1 mm (length × width × thickness) (*n* = 10).

Prior to manufacturing the specimens, a positioning guide measuring 10 × 1 × 1 mm was prepared from Filtek Z350 XT™ (3M/ESPE) composite resin and carefully measured using a digital caliper. This guide was prepared to assist with positioning a razor blade in the center of the metal mold, standardizing this positioning, and facilitating the production of a notch (slit) 0.9 mm deep in each sample. The razor blade was fixed in the metal mold using sticky wax.

After fixing the razor blade, the resin composite positioning guide was removed, and the hole in the mold was filled with experimental composite; this mold-composite was pressed using polyester sheets and glass panes on both sides of the mold to allow the removal of excess material and standardize the thickness. After filling the mold with composite, the upper layer of material was photoactivated using the curing device.

The active tip of the curing device was 8 mm in diameter. Thus, the tip was kept 1 mm from the experimental resin at the moment of photoactivation to allow the curing light to reach the entire specimen (which was 10 mm wide). This arrangement allowed the distance between the active tip of the device and the material to ensure that the light would reach all of the material. The power density was 16 J/cm^2^.

Then, one-half of the specimens were placed in an oven for thermal treatment, as previously described. Next, all specimens were stored at 37°C for 24 hours. Following storage, a mechanical testing machine (model 5565, Instron, Canton, Ohio, USA) was used to subject the specimens to loading with the aid of a device for the 3-point bend test. The supports were separated by 8 mm. Specimens were positioned such that the fabricated notch was opposite the point generating the applied load. The loading speed was 0.13 mm/min.

After obtaining the data for failure loads, the fracture surfaces on the specimens were analyzed using a CCD camera (model SZ61, Olympus, Tokyo, Japan) with up to 90x magnification coupled to a stereomicroscope. The notch depth (*a*), width (*w*), and thickness (*b*) of each specimen were measured from the obtained images using the software ImageJ (National Institutes of Health, Bethesda, Maryland, USA). The arithmetic mean of 6 notch depth measurements, 3 from each fracture surface, was used to calculate “*a*.”

*K*_IC_ (in MPa·m^0.5^) was calculated using the following equation:

(2) $$\begin{array}{c} K_{IC}=\left[\;\frac{P\times S}{b\times w^{1,5}}\;\right]\times f\left(\;\frac{a}{w}\;\right), \end{array}$$

where *P* is the maximum failure load (N); *S* is the distance between supports (m); *b* and *w* were converted to meters; and *f*(*a*/*w*) was calculated using the following equation:

(3) $$\begin{array}{c} f\left(\;\frac{a}{w}\;\right)=3\sqrt{\left(\frac{a}{w}\right)}\left(\;\frac{\text{1,99}-\left(a/w\right)\times \left[1-\left(a/w\right)\right]\times \left[{\text{2,15}-\text{3,93}\left(a/w\right)+\text{2,7}\left(a/w\right)}^{2}\right]}{2\times \left[1+2\left(a/w\right)\right]\times {\left[1-\left(a/w\right)\right]}^{3/2}}\;\right). \end{array}$$

### 2.5. Flexural Strength and Elastic Modulus

The specimens were manufactured in the shape of a bar (*n* = 10) from a steel mold that had an internal cavity measuring 10 × 2 × 1 mm. The method for manufacturing the specimens was similar to that used for the fracture toughness test in terms of filling the internal mold cavity and pressing using Mylar strips and glass panes on both sides of the mold and curing. After manufacturing the molds, half of the specimens were subjected to postcuring thermal treatment, and both the thermal-treated and control specimens were immediately stored at 37°C for 24 hours.

The flexural strength test was performed using a mechanical testing machine (Instron, model 5565) followed by the 3-point bend test after 24 hours. A 1,000 Newton load cell and a 0.5 mm/min loading speed were used. The specimens were centered in the machine using an aluminum guide, and the supports were separated by 8 mm. The support bars and loading bar on the machine were cylindrical with a 2 mm diameter. Before conducting the test, each specimen was measured using a digital caliper accurate to 1 *μ*m (Mitutoyo, Tokyo, Japan) such that the specimen width and height could be entered in the following formula:

(4) $$\begin{array}{c} FS=\frac{3lf}{2bh^{2}}, \end{array}$$

where FS is the flexural strength (MPa); *f* is the failure load (N); *l* is the distance between supports (10 mm); *b* is the specimen width (mm); and *h* is the specimen height (mm).

At the moment of testing, it was also possible to obtain a linear portion of the load × displacement curve, which was used to calculate the EM. The following formula was used:

(5) $$\begin{array}{c} EM=\frac{C\times L^{3}}{4\times l\times h^{3}\times d}\times 10^{3}, \end{array}$$

where EM is the elastic modulus (GPa); *C* is the load recorded (N); *L* is the span between supports (mm); *l* is the specimen width (mm); *h* is the specimen height (mm); and *d* is the displacement related to *C*.

### 2.6. Statistical Analysis

The data were initially analyzed to test for a normal distribution, followed by analysis of sample homoscedasticity. The sample was normally distributed and homoscedastic for analyses of degree of conversion, fracture toughness, and flexural strength, thus allowing the application of analysis of variance (ANOVA) with 3 factors (monomer, filler, and thermal treatment) and Tukey's post hoc test with a 5% overall level of significance (*p* < 0.05). In contrast, the elastic modulus data were not normally distributed or homoscedastic; therefore the Kruskal-Wallis test was applied, and the means were compared using Tukey's post hoc test (*p* < 0.05).

## 3. Results

The triple interaction among the factors was not statistically significant. Tables 2 and 3 show the mean and standard deviation for the degree of conversion, fracture toughness, flexural strength, and elastic modulus for each factor evaluated (monomer, thermal treatment, and filler). Assessment of the monomer variance factor revealed that the greatest values for degree of conversion, fracture toughness, flexural strength, and elastic modulus were obtained with the equimolar composition of BisGMA and TEGDMA. For the inorganic content variance factor, the amount of filler positively affected fracture toughness, flexural strength, and elastic modulus, and a decrease was observed in the degree of conversion of the material with 70 wt%. For the thermal treatment variance factor, it was evident that such treatment increased the degree of conversion, fracture toughness, flexural strength, and elastic modulus (*p* < 0.001).

Statistical analysis of factor interactions revealed a significant monomer × thermal treatment interaction for degree of conversion (*p* < 0.001), while the interactions monomer × filler (*p* = 0.001) and filler × thermal treatment (*p* < 0.001) were significant for EM. For the monomer and filler interactions, the greatest elastic modulus values were achieved with a 1 : 1 BisGMA : TEGDMA ratio and 70 wt%.

## 4. Discussion

The null hypotheses of this study were rejected since the factors of monomer composition, inorganic content, and thermal treatment significantly affect the evaluated properties.

BisGMA and TEGDMA monomers were used in the present study because they are more commonly found in dental composites. Our results indicated that the composition of the organic matrix influenced the degree of conversion, and the highest conversion (85%) values were observed with a 1 : 1 BisGMA : TEGDMA ratio. It is believed that a greater concentration of TEGDMA (in weight) is favorable because this diluent helps reduce the viscosity of BisGMA, increasing the molecular mobility and thereby facilitating polymerization. Additionally, the decrease of TEGDMA reduced the degree of conversion due to the more rigid structure and higher viscosity of the BisGMA molecules [27]. The study by Floyd & Dickens [28] has shown that increasing the diluent monomer concentration not only increases the conversion but also reduces the residual monomer fraction and increases network reticulation [28], which could affect the mechanical properties of the material. The amount of filler should also be considered an important factor to determine the degree of conversion. The highest filler concentration (70%) resulted in a lower degree of conversion values, which can most likely be explained by two main reasons: an increase in viscosity that consequently decreased the polymerization reaction and the higher light scattering performed by the load fillers.

The degree of conversion was higher after postcuring thermal treatment for all evaluated materials. It is known that both temperature and thermal treatment duration significantly affect material conversion [29]. Heating a composite to a temperature above its glass transition temperature (approximately 160°C) enables rotation/movement of unreacted radicals, thereby permitting an increase in conversion [18, 30] and greater relaxation of stresses induced during polymerization [31] resulting in better mechanical properties. The postcuring thermal treatment protocol described for the present study consisted of maintaining the specimens at 170°C for 10 min because this temperature is above the *T*_*g*_ and below that which could degrade the material [19, 20, 32]. Previous studies have already indicated that this protocol promotes an increase in conversion and improved mechanical properties [19, 32], and these findings also agree with those from another study [18]. A significant monomer × thermal treatment interaction for degree of conversion was observed. The administration of thermal treatment promoted a greater degree of conversion for all monomer compositions; however, it was more pronounced with the 7 : 3 BisGMA : TEGDMA ratio monomer composition, possibly due to the higher number of residual monomers in these materials.

The mechanical properties of fracture toughness, flexural strength, and elastic modulus demonstrated similar behavior according to the factors evaluated: the polymeric matrices with a 1 : 1 BisGMA : TEGDMA ratio presented higher fracture toughness, flexural strength, and elastic modulus than the 7 : 3 BisGMA : TEGDMA ratio; the values of these properties increased in accordance with the filler content; and the thermal treatment was able to increase all the mechanical properties' values. The higher concentration of TEGDMA in the 1 : 1 ratio compared to the 7 : 3 ratio resulted in greater fracture toughness, flexural strength, and elastic modulus; these data are in accordance with those obtained in other studies [9, 17, 33] and could be a consequence of the higher degree of conversion in these matrices. Regarding the effect of the filler amount in the mechanical properties, there are direct relationships between the filler concentration and the fracture toughness, flexural strength, and elastic modulus. These relationships have been reported by other studies [6, 12, 34]. The effect of filler on the fracture toughness possibly occurs because the cracks propagate through the polymer matrix around filler particles, and the increase in the inorganic content promotes greater compression of the material, allowing a reduction of the force acting on these particles with consequent minimization of crack propagation [12].

The application of thermal treatment affected fracture toughness, flexural strength, and elastic modulus, which agrees with prior studies [18–20], which could be explained by an increase in degree of conversion and stress relief caused by an increase beyond the *T*_*g*_ of the composites, suggesting that this is a relevant mechanism for improving their mechanical properties [19, 20].

Regarding the elastic modulus, significant monomer × filler and filler × thermal treatment interactions were observed. In a 1 : 1 BisGMA : TEGDMA ratio, the filler concentration was able to promote a higher increase in the elastic modulus at 50% to 70%, whereas in the 7 : 3 BisGMA : TEGDMA ratio, the effect of the filler was more pronounced in the 30% and 50% filler concentrations. For the filler × thermal treatment interaction, it was observed that the effect of the filler to increase the elastic modulus was more pronounced in the specimens that did not receive the thermal treatment.

Based on the results obtained, it can be concluded that the polymeric matrix composition and filler are important factors that should be evaluated concomitantly during the development of dental composites in order to reach composites with high conversion and high mechanical properties. In this study, the equimolar BisGMA : TEGDMA ratio was able to provide both better conversion and higher mechanical properties. Although the use of the 70% filler concentration decreased the conversion when compared to the 30 and 50 wt% groups, this decrease was not enough to decrease the mechanical properties, therefore being a good choice in the design of materials. In addition, the application of postcuring resulted in improvement of the studied properties. The thermal treatment proposed in this study may represent an efficient, practical, and low-cost alternative to the current indirect systems of dental composites. Although the indirect restoration with composite resin shows mechanical and aesthetic properties lower than the ceramics in general, they still have their indications, for example, for patients with parafunctional habits, as a low-cost treatment in emergent countries, as provisional treatment, and as antagonist of total and partial denture.

## Figures and Tables

**Table 1:** Composition of experimental composites.

BisGMA (mol%)	TEGDMA (mol%)	Camphorquinone (mol%)	Amine (mol%)	Filler (% weight)
50	50	2	2	30
50	50	2	2	50
50	50	2	2	70
70	30	2	2	30
70	30	2	2	50
70	30	2	2	70

**Table 2:** Means ± standard deviations for degree of conversion (DC), fracture toughness (*K*_IC_), flexural strength (FS), and elastic modulus (EM) for BisGMA : TEGDMA molar ratio and thermal treatment factors. For each property, the same letter indicates that there is no statistically significant difference (*p* > 0.05).

	Molar ratio of BisGMA : TEGDMA		Thermal treatment	
	1 : 1	7 : 3	No	Yes
DC (%)	85 ± 5.2^a^	80 ± 7.1^b^	77 ± 2.0^b^	89 ± 2.0^a^
*K*_(IC)_	1.46 ± 0.17^a^	1.20 ± 0.17^b^	1.26 ± 0.21^b^	1.40 ± 0.20^a^
FS (MPa)	152 ± 15^a^	140 ± 15^b^	138 ± 15^b^	155 ± 14^a^
EM (GPa)	6.1 ± 2.0^a^	5.4 ± 1.8^b^	5.3 ± 1.8^b^	6.1 ± 1.9^a^

**Table 3:** Means ± standard deviations for degree of conversion (DC), fracture toughness (*K*_IC_), flexural strength (FS), and elastic modulus (EM) for filler concentration factor. For each property, the same letter indicates that there is no statistically significant difference (*p* > 0.05).

	Filler content		
	30%	50%	70%
DC (%)	83 ± 7.0^a^	83 ± 7.0^a^	82 ± 7.0^b^
*K*_(IC)_	1.20 ± 0.20^c^	1.34 ± 0.19^b^	1.44 ± 0.20^a^
FS (MPa)	134 ± 16^c^	147 ± 13^b^	157 ± 11^a^
EM (GPa)	4.0 ± 0.3^c^	5.1 ± 0.9^b^	8.1 ± 0.8^a^
